# *Gnetum montanum* extract attenuates lipopolysaccharide induced acute lung inflammation through Nrf2 and heme oxygenase 1 mediated redox modulation in macrophages

**DOI:** 10.1038/s41598-026-50370-z

**Published:** 2026-04-24

**Authors:** Duc-Vinh Pham, Hong-Linh Tran, Thu-Hang Nguyen, Hong-Nhung Bui, Hai-Nam Nguyen, Thanh Nguyen Le, Thi Hien Pham, Thuy-Duong Nguyen

**Affiliations:** 1 Department of Pharmacology, Hanoi University of Pharmacy, 13-15 Le Thanh Tong, Cua Nam, Hanoi, Vietnam; 2 Department of Medicinal Chemistry, Hanoi University of Pharmacy, 13-15 Le Thanh Tong, Cua Nam, Hanoi, Vietnam; 3 Department of Analytical Chemistry and Standardization, National Institute of Medicinal Materials, 3B Quang Trung, Cua Nam, Hanoi, Vietnam

**Keywords:** Acute lung injury, *Gnetum montanum*, Inflammation, Nrf2, Oxidative stress, Biochemistry, Diseases, Drug discovery, Immunology, Plant sciences

## Abstract

The Nrf2/HO-1 pathway is a promising therapeutic target for inflammatory disease management. Stilbene derivatives can activate this pathway and exert potent anti-inflammatory effects. *Gnetum montanum* Markgr., a stilbene-rich medicinal plant traditionally used in Vietnam, China, and other Asian countries for the treatment of inflammatory disorders, has not been systematically investigated for its anti-inflammatory properties or underlying mechanisms. Our study evaluated the protective effects of a stilbene-rich *G. montanum* extract (SGME) against inflammatory responses in vitro and in vivo. In lipopolysaccharide (LPS)-activated raw 264.7 macrophages, SGME significantly suppressed the expression and production of pro-inflammatory mediators by inhibiting NF-κB activation. In bone marrow-derived macrophages, SGME effectively prevented polarization toward the pro-inflammatory M1 phenotype. Mechanistically, SGME promoted Nrf2 nuclear translocation, leading to the upregulation of HO-1 and other Nrf2 target genes, attenuating LPS-induced oxidative stress in macrophages. Both Nrf2 knockdown and pharmacological inhibition significantly diminished these protective effects, confirming Nrf2’s pivotal role. Notably, in an oropharyngeal LPS-induced acute lung inflammation model, SGME reduced leukocyte infiltration, suppressed excessive cytokine production, and alleviated oxidative stress, accompanied by increased expression of Nrf2-dependent antioxidant genes, including *Cat*, *Sod2*, and *Hmox1*. These findings demonstrate that SGME exerts potent anti-inflammatory effects via Nrf2 pathway activation, warranting further studies to evaluate its therapeutic potential in inflammation-related diseases.

**Supplementary Information:**

The online version contains supplementary material available at 10.1038/s41598-026-50370-z.

## Introduction

Acute and chronic inflammation are essential physiological responses that contribute to the pathogenesis of a wide range of diseases, including infections, rheumatic disorders, inflammatory bowel disease, and metabolic and cardiovascular conditions^1^. For decades, anti-inflammatory therapy has relied primarily on two classical drug classes: non-steroidal anti-inflammatory drugs and glucocorticoids^2^. However, their clinical utility is often limited by adverse effects and, in some cases, limited efficacy, as these agents do not address the underlying drivers of inflammation^3^. Recently, novel therapeutic strategies directed against key pro-inflammatory cytokines, such as tumor necrosis factor (TNF)-α and interleukin (IL)-1β, have demonstrated efficacy in selected inflammatory disorders. However, all currently available anti-cytokine biologics are protein-based and subject to the inherent limitations of protein therapeutics^4^. These challenges have led to growing interest in plant-derived agents, which often act through multiple complementary pathways and may offer favorable tolerability profiles^5^.

Inflammation-associated lung injury is a representative condition in which dysregulated inflammatory signaling and oxidative stress closely interact to amplify tissue damage. In the lung, exposure to inflammatory stimuli triggers macrophage activation, cytokine release, and leukocyte recruitment through multiple pathways, including NF-κB, MAPK, and JAK-STAT^6^. Concomitantly, excessive production of reactive oxygen species (ROS) further aggravates this response. Indeed, ROS act not only as damaging oxidants but also as signaling mediators^7^. ROS can activate MAPK signaling by oxidizing thioredoxin and releasing ASK1, thereby promoting downstream JNK/p38 activation, and by oxidatively inactivating phosphatases such as protein tyrosine phosphatases and MAPK phosphatases (PTPs/MKPs) that normally terminate MAPK signaling^8,9^. In parallel, ROS can potentiate NF-κB signaling in inflammatory settings, at least in part by enhancing IKK activation and/or redox-dependent IκBα phosphorylation, leading to IκBα inactivation or degradation, NF-κB nuclear translocation, and transcription of pro-inflammatory genes^10^. In the lung, this ROS-driven amplification disrupts epithelial-endothelial barrier integrity and promotes pulmonary edema and tissue damage, thereby accelerating the progression of acute inflammatory lung injury^11^.

Among endogenous cytoprotective systems that counteract inflammatory and oxidative damage, Nrf2-dependent antioxidant signaling, including the Nrf2/HO-1 axis, has emerged as an important protective pathway^12^. Under basal conditions, Nrf2 remains sequestered in the cytoplasm through its interaction with Kelch-like ECH-associated protein 1 (Keap1). In response to oxidative stress, Nrf2 dissociates from Keap1 and translocates to the nucleus, where it binds antioxidant response elements in the promoters of target genes^13^. These include heme oxygenase-1 (HO-1/HMOX1), superoxide dismutase (SOD), catalase (CAT), and NAD(P)H: quinone oxidoreductase 1 (NQO1)^14^. Beyond its antioxidant functions, Nrf2 exerts potent anti-inflammatory effects^15^. By inducing antioxidant gene expression, Nrf2 reduces intracellular ROS levels, attenuating ROS-driven inflammatory amplification. In addition, Nrf2 suppresses NF-κB activation, a central pathway in pro-inflammatory cytokine production^16^. Several studies indicate that these anti-inflammatory effects are largely mediated through HO-1 induction^17^. Indeed, HO-1 expression is markedly upregulated under stress conditions to mitigate oxidative injury^18^. Furthermore, HO-1 regulates inflammatory responses through multiple mechanisms. In particular, its metabolites, such as carbon monoxide, reduce the secretion of pro-inflammatory mediators by modulating the NF-κB and MAPK signaling pathways^19,20^. Therefore, activation of Nrf2/HO-1 represents one of several complementary approaches for controlling inflammation-associated disorders.

Natural products capable of activating Nrf2 signaling have attracted considerable attention as potential modulators of inflammatory responses. Several phytochemicals, including stilbene derivatives, have shown protective effects in inflammatory and oxidative stress-related models through activation of the Nrf2/HO-1 axis. For example, resveratrol and oxyresveratrol activate Nrf2, at least in part through modulation of Keap1, and Nrf2/HO-1 signaling has been implicated in the protective effects of resveratrol in inflammatory disorders^21–23^. Likewise, isorhapontigenin has been reported to attenuate LPS-induced acute lung injury in mice by promoting Nrf2 nuclear translocation^24^. Collectively, these findings suggest that stilbene-rich medicinal plants may represent promising candidates for controlling oxidative and inflammatory lung damage.

In this context, *Gnetum montanum* Markgr., a climbing plant of the Gnetaceae family native to India, Southeast Asia, and China^25^, is a promising candidate for further investigation. In traditional Vietnamese medicine, its lianas and roots have been used to treat inflammation-associated conditions, including rheumatism, gouty arthritis, bone pain, menstrual irregularities, and snakebites. Importantly, its therapeutic interest is supported not only by ethnopharmacological use but also by its phytochemical composition. Studies conducted in China and Vietnam have identified diverse bioactive constituents in *G. montanum*, including stilbenoids, flavonoids, lignans, phenolics, and alkaloids^26,27^. Previous investigations have primarily highlighted its antioxidant, anticancer, and xanthine oxidase inhibitory activities in crude extracts and isolated constituents from stems and roots^28–30^, indicating a pharmacologically relevant profile linked to redox regulation. Notably, stilbenes have been reported as major constituents of *G. montanum* extracts in these studies, providing a plausible mechanistic basis for its potential anti-inflammatory activity through modulation of Nrf2-associated cytoprotective signaling.

Despite its traditional use and the presence of bioactive constituents implicated in redox and inflammatory regulation, the anti-inflammatory potential of *G. montanum* extract has not been systematically evaluated in inflammation-associated lung injury. Moreover, it remains unclear whether the Nrf2/HO-1 pathway contributes to its effects. To address this knowledge gap, we hypothesized that *G. montanum* extract exerts anti-inflammatory effects, at least in part, through Nrf2/HO-1-mediated redox modulation in macrophages. Accordingly, we investigated the anti-inflammatory properties of *G. montanum* extract in Raw 264.7 and bone marrow-derived macrophages and in a murine model of LPS-induced lung inflammation, with a particular focus on the contribution of the Nrf2/HO-1 axis.

## Results

### SGME attenuates LPS-induced inflammatory responses in raw 264.7 macrophages

**Fig. 1:**
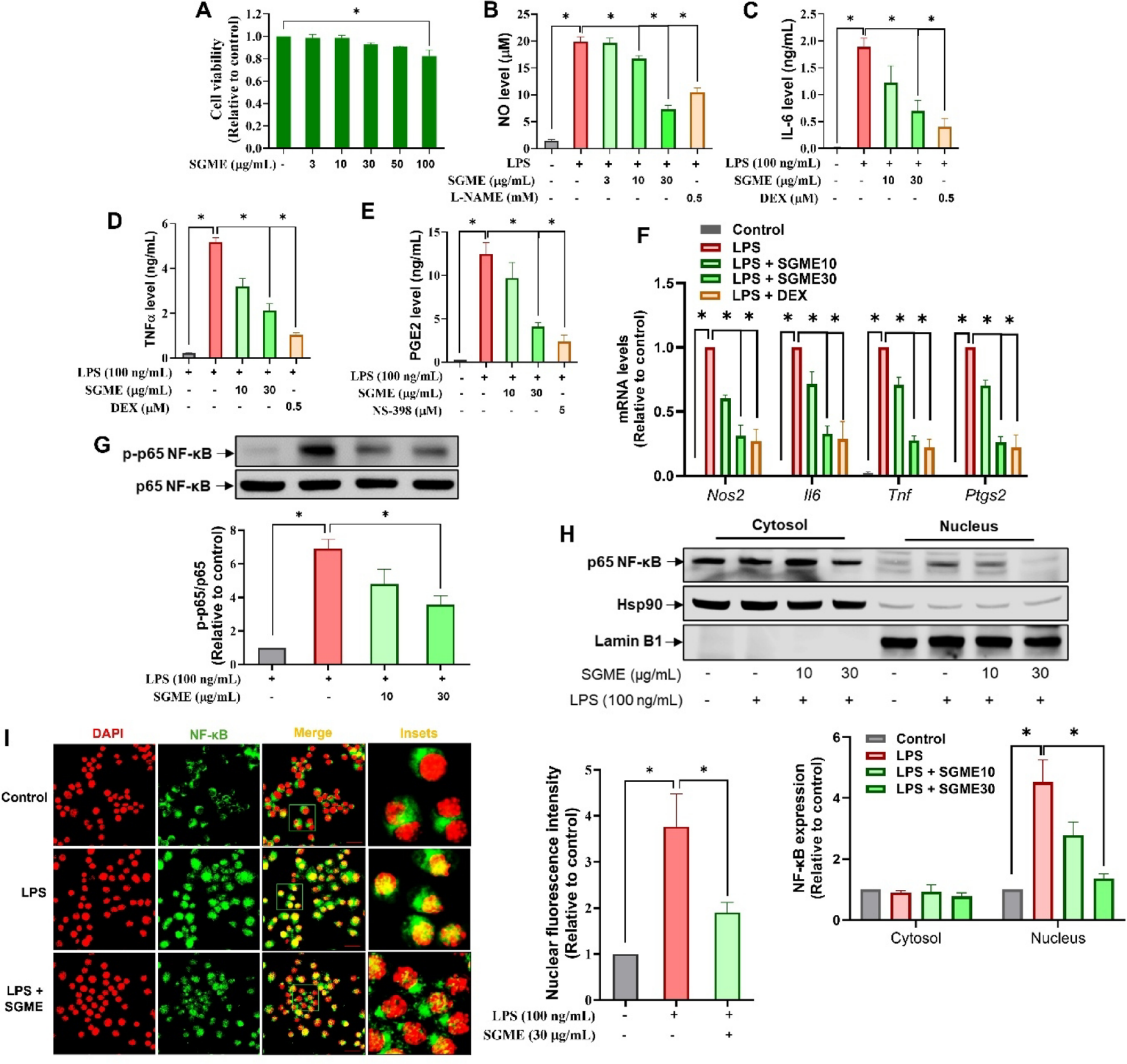
Effects of SGME against LPS-induced inflammatory responses in Raw 264.7 macrophages. (**A**) Raw 264.7 macrophages were incubated with increasing concentrations of SGME for 24 h. Cell viability was then examined by the MTT assay. (**B**–**F**) Cells were treated with SGME for 3 h and activated with LPS (100 ng/mL) for an additional 24 h (**B**–**E**) or 6 h (**F**). The levels of nitrite/nitrate (**B**), IL-6 (**C**), TNF-α (**D**), and prostaglandin E_2_ (PGE2) (**E**) were measured in culture medium collected at the end of the treatment period. (**F**) The mRNA levels of *Nos2*,* Il6*,* Tnf*, and *Ptgs2* were measured by RT-qPCR assays. (**G** and **H**) Cells were treated with SGME at concentrations of 10 (SGME10) and 30 (SGME30) µg/mL for 3 h and then with LPS (100 ng/mL) for 60 min. The protein levels of p-p65 NF-κB and total p65 NF-κB were determined in cellular lysates and nuclear/cytoplasmic fractions, respectively, by western blot. Representative images and blot quantification are shown. Uncropped blot images were provided as supplementary material. (I) Subcellular distribution of p65 NF-κB was analyzed by immunocytochemistry (red: nuclei; green: p65 NF-κB; scale bar: 20 μm). The nuclear fluorescence intensity of p65 NF-κB was calculated using the ImageJ application. * denotes *p* < 0.05; *n* = 3.–

The anti-inflammatory effect of the *G. montanum* extract (SGME) was initially investigated in LPS-activated Raw 264.7 macrophages. To select appropriate extract concentrations for subsequent experiments, an MTT assay was employed to evaluate the effect of SGME on cell viability. As shown in Fig. 1A, SGME at 100 µg/mL significantly reduced cell viability; however, this effect was not observed at lower concentrations. LPS is thought to trigger inflammatory responses in macrophages by inducing the production and release of pro-inflammatory mediators. Herein, we also found that LPS stimulation resulted in increases in the production of pro-inflammatory mediators such as nitric oxide (NO), IL-6, TNF-α, and prostaglandin E2 (PGE2) in Raw 264.7 macrophages (Fig. 1B–E). In contrast, pretreatment with SGME attenuated the production of these molecules in a dose-dependent manner. In particular, SGME at 30 µg/mL reduced the secretion of NO, IL-6, TNF-α, and PGE2 by more than 50%. SGME at 10 µg/mL, but not at 3 µg/mL, also significantly suppressed NO production (Fig. 1B). Therefore, SGME at 10 and 30 µg/mL was used for subsequent assays in this study. Consistent with these observations, RT-qPCR analysis showed that SGME inhibited the upregulation of genes encoding iNOS, an enzyme mainly responsible for NO production in response to pro-inflammatory stimuli, as well as IL-6, TNF-α, and COX-2, a rate-limiting enzyme in PGE2 synthesis (Fig. 1F). Given that the expression of pro-inflammatory genes is largely controlled by the transcription factor NF-κB, we next examined the effect of SGME on the phosphorylation and nuclear translocation of NF-κB. Western blot analysis indicated that SGME substantially reduced the level of phospho-p65 (Fig. 1G). Additionally, SGME suppressed nuclear p65 levels but did not affect cytoplasmic p65 levels (Fig. 1H). Likewise, immunocytochemistry (ICC) showed that NF-κB accumulated in the nucleus in response to LPS stimulation, whereas SGME prevented LPS-induced nuclear translocation of NF-κB (Fig. 1I). Taken together, these results indicate that SGME attenuated the inflammatory responses in LPS-stimulated macrophages by decreasing the expression and production of pro-inflammatory mediators via suppression of NF-κB activation.

### SGME inhibits the M1 macrophage polarization in bone marrow-derived macrophages

**Fig. 2:**
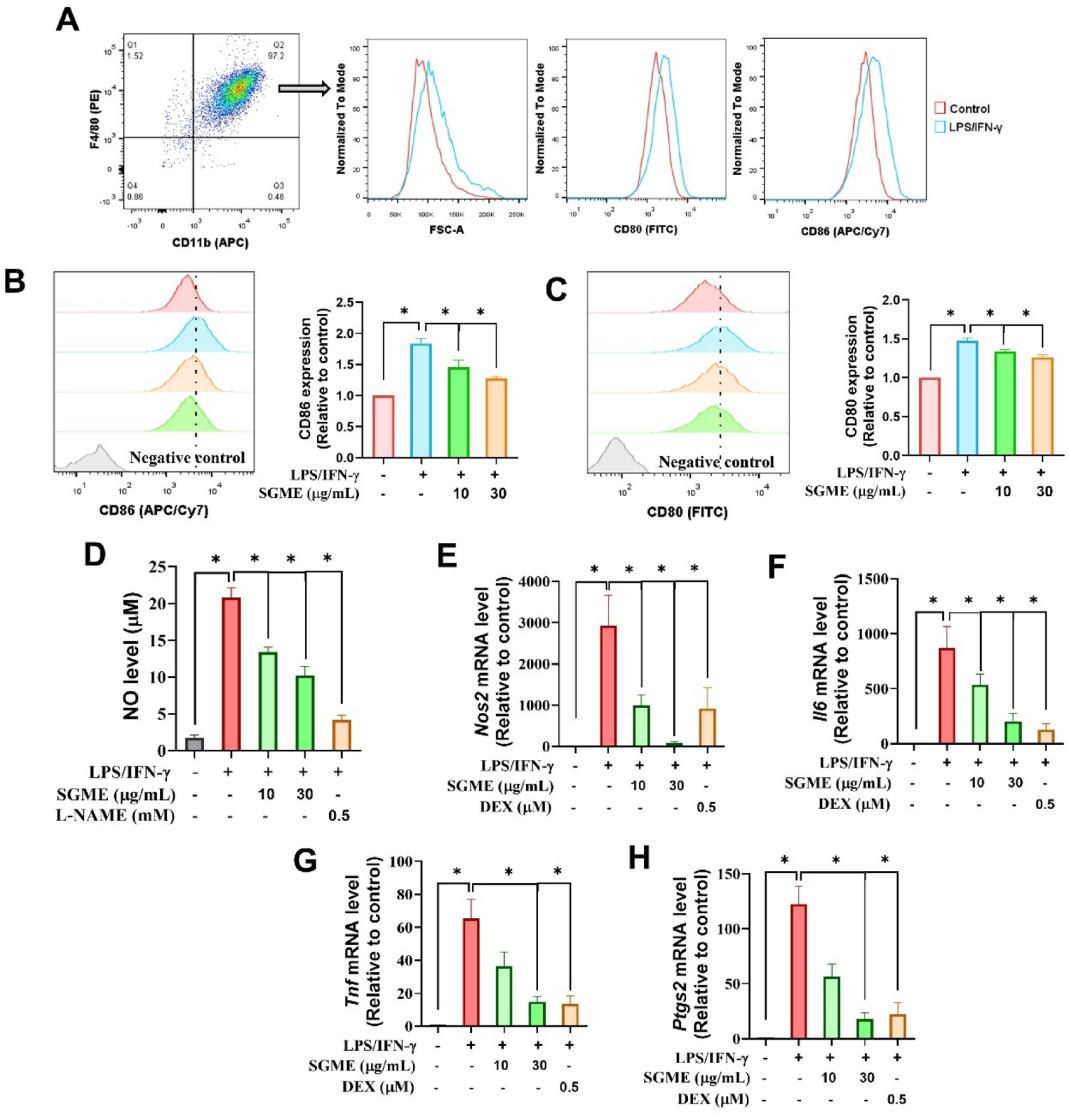
Effects of SGME on the polarization of bone marrow-derived macrophages (BMDMs). Bone marrow-derived monocytes were first differentiated into macrophages by incubating cells with GM-CSF for 7 days. BMDMs were then cultured in medium containing LPS/IFN-γ (100/10 ng/mL) for 24 h. (**A**) Phenotype characterization of BMDMs with or without LPS/IFN-γ exposure using flow cytometry analysis. An illustration of the gating strategy is provided in Supplementary Fig. S1. **(B**–**H**) BMDMs were treated with SGME or positive controls including L-NAME and dexamethasone (DEX) 3 h before polarization with LPS/IFN-γ for 24 h. (**B** and **C**) The expression of M1 markers including CD86 (**B**) and CD80 (**C**) in live cells was examined by flow cytometry analysis. (**D**) Nitrite/nitrate levels were quantified in culture medium. (**E**–**H**) The mRNA levels of genes encoding iNOS (*Nos2*), IL-6 (*Il6*), TNF-α (*Tnf*), and COX-2 (*Ptgs2*) were determined using RT-qPCR assays. * denotes *p* < 0.05; *n* = 3.––

During the acute inflammatory response, macrophages are recruited to inflamed tissues and polarized into the pro-inflammatory M1 phenotype. We next examined whether SGME modulates this polarization process of macrophages under an inflammatory environment. Herein, bone marrow-derived monocytes were first differentiated into M0 macrophages, followed by culturing in medium containing LPS and IFN-γ to induce M1 polarization. Flow cytometry analysis of F4/80 and CD11b confirmed that the differentiated cell population consisted predominantly of macrophages (97.2%) (Fig. 2A). Upon LPS/IFN-γ stimulation, BMDMs exhibited an increased forward scatter (FSC-A), consistent with cellular enlargement, together with a clear rightward shift in CD80 and CD86 expression compared with the control group, confirming successful induction of an M1-like phenotype. Treatment with SGME inhibited the conversion of M0 into M1 macrophages, as corroborated by decreased expression levels of CD86 (Fig. 2B) and CD80 (Fig. 2C) in LPS/IFN-γ stimulated macrophages. Consistent with the M1 phenotype, BMDMs increased the production of NO (Fig. 2D) and the expression of pro-inflammatory genes including *Nos2*,* Il6*,* Tnf*, and *Ptgs2* (Fig. 2E–H) in response to LPS/IFN-γ; however, SGME significantly abrogated these effects of LPS/IFN-γ. Collectively, these observations indicated that SGME prevents M0 macrophages from polarizing into the M1 pro-inflammatory phenotype.

### SGME activates the Nrf2/HO-1 signaling pathway in macrophages

**Fig. 3:**
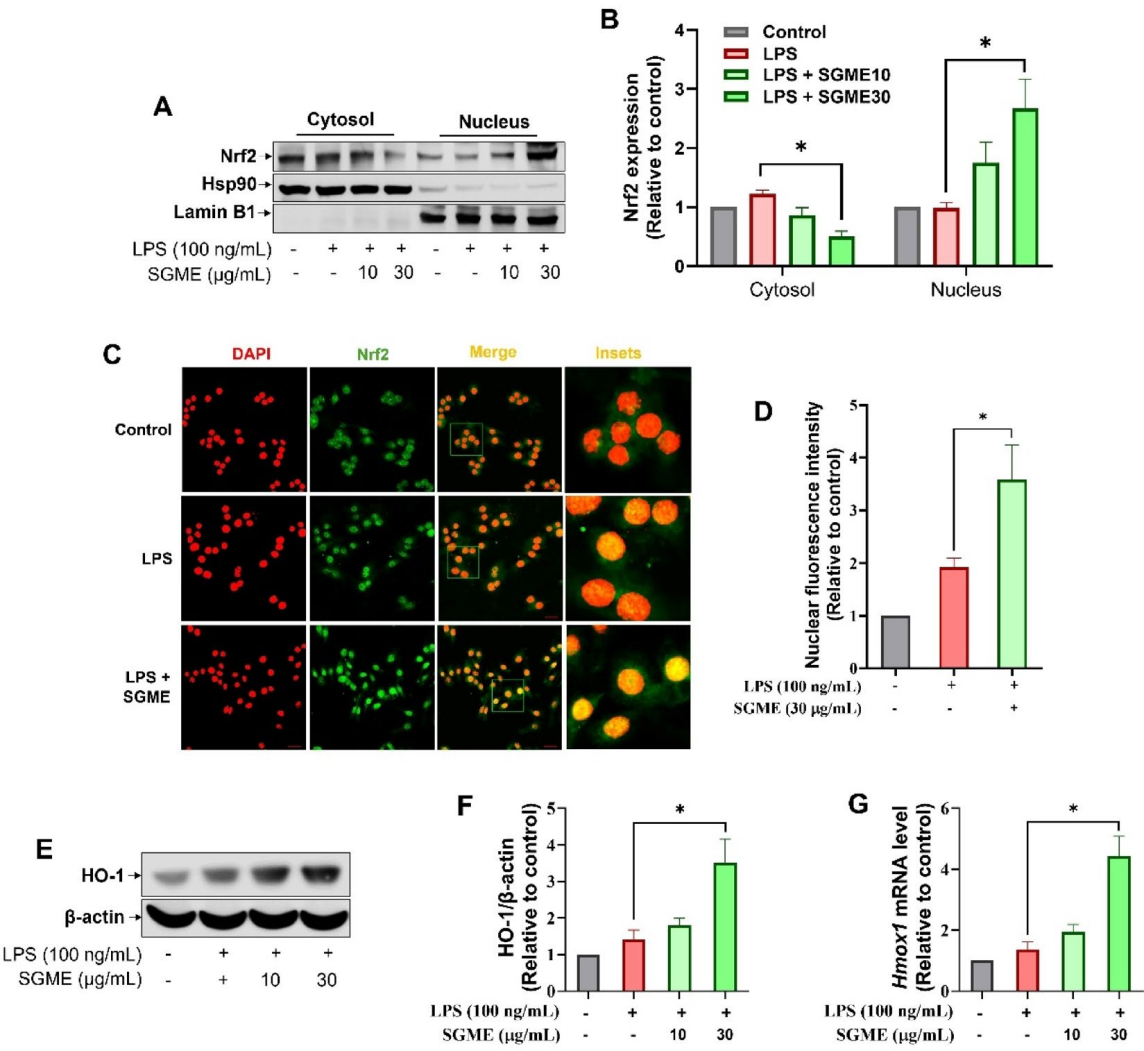
Modulatory effects of SGME on the Nrf2/HO-1 signaling pathway. (**A**–**D**) Raw 264.7 macrophages were pretreated with SGME for 3 h and then stimulated with LPS for 1 h. (**A** and **B**) Protein levels of Nrf2 in nuclear and cytoplasmic lysates were examined by western blot. (**C** and **D**) Nrf2 was labeled by immunostaining. Representative images showed the distribution of Nrf2 (green) in relation to nuclei (red); the bar graph depicted relative nuclear fluorescence intensity of Nrf2 calculated by ImageJ; scale bar: 20 μm. (**E**–**G**) After treatment with SGME for 3 h, Raw 264.7 macrophages were treated with LPS for 3 h. Protein and mRNA levels of HO-1 were measured by western blot and RT-qPCR. * denotes *p* < 0.05; *n* = 3.–

To explore the molecular mechanisms underlying the anti-inflammatory effects of SGME, we tested the hypothesis that SGME modulates the Nrf2/HO-1 signaling pathway, which has been postulated to play a key role in controlling inflammatory responses. To this end, we first examined the effects of SGME on the nuclear translocation of Nrf2 in LPS-activated macrophages. The results showed that SGME promoted the translocation of Nrf2 to the nucleus, as evidenced by increased protein levels of Nrf2 in nuclear lysates but decreased levels in cytoplasmic lysates (Fig. 3A and B). The ICC analysis also indicated that SGME enhanced the distribution of Nrf2 into the nucleus (Fig. 3C). We next evaluated the effects of SGME on Nrf2 target genes and found that SGME enhanced the protein expression of HO-1 in a dose-dependent manner (Fig. 3D and E). Moreover, SGME upregulated the expression of HO-1 at the transcriptional level (Fig. 3F). In addition to *Hmox1*, SGME also increased the expression of other Nrf2-regulated genes such as *Cat* and *Sod2* (Supplementary Fig. S2). Overall, these results suggested that SGME activates the transcription of Nrf2-dependent genes by inducing the nuclear translocation of this transcription factor.

### Activation of Nrf2/HO-1 by SGME leads to suppression of ROS production in macrophages

**Fig. 4:**
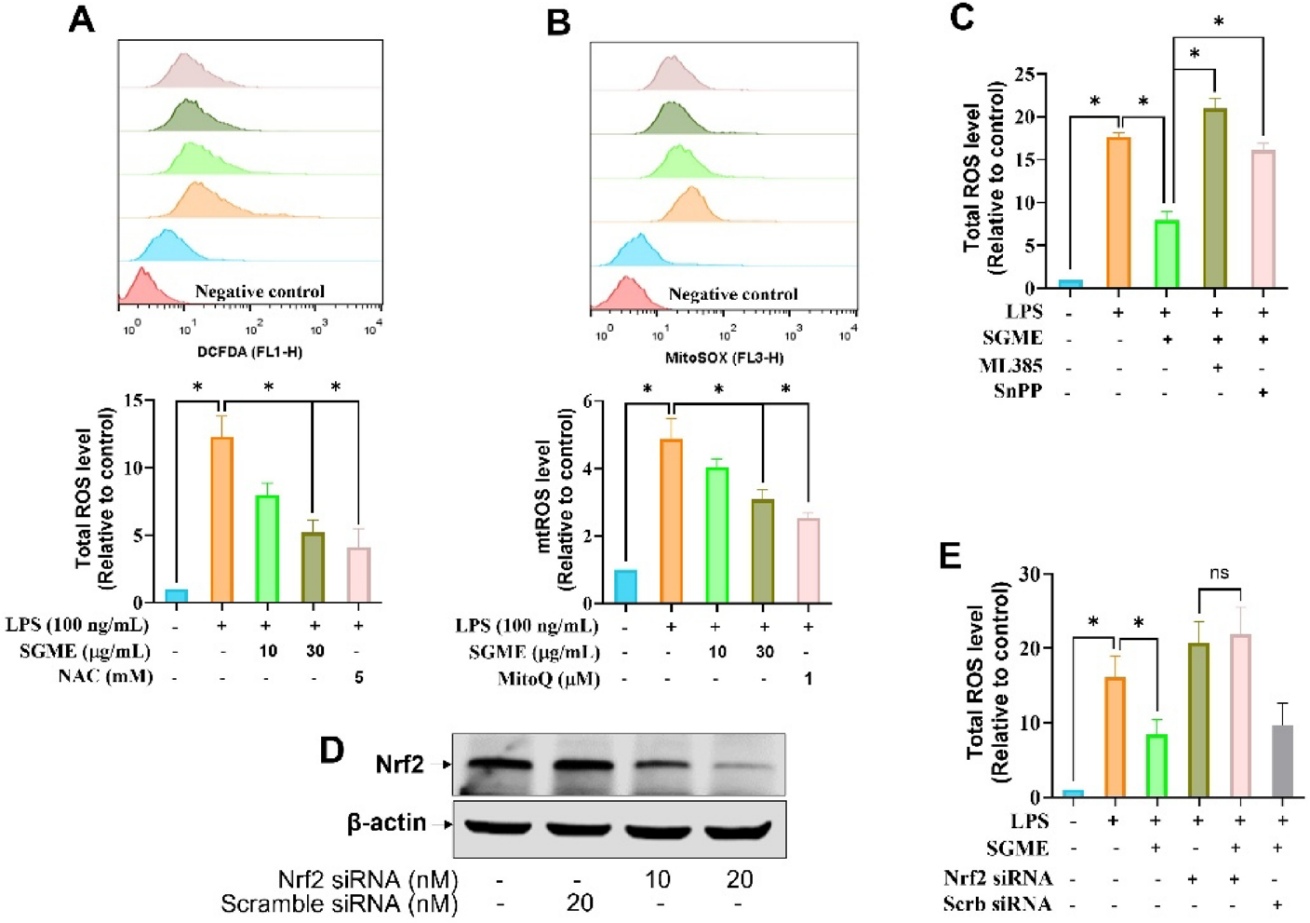
Roles of Nrf2/HO-1 in modulation of ROS production by SGME. (**A **and** B**) Raw 264.7 macrophages were pretreated with SGME, N-acetylcysteine (NAC), or mitoquinone mesylate (mitoQ) for 3 h, followed by stimulation with LPS for 24 h. Total cellular and mitochondrial ROS (mtROS) were detected by CM-H2DCFDA and MitoSOX Red, respectively, using flow cytometry. (**C**) Cells were sequentially incubated with SnPP (10 µM), a pharmacological inhibitor of HO-1, or ML385 (10 µM), a pharmacological inhibitor of Nrf2, for 1 h, SGME (30 µg/mL) for 3 h, and LPS (100 ng/mL) for 24 h before cellular ROS levels were determined. (**D **and** E**) The Nrf2 gene was knocked down by transfection of cells with a specific siRNA targeting Nrf2. (D) The transfection efficiency was examined after 24 h of transfection. (E) After siRNA transfection for 24 h, cells were treated with SGME (30 µg/mL) for 3 h and then with LPS (100 ng/mL). Total ROS levels were measured at 24 h after LPS stimulation. * denotes *p* < 0.05; ns: statistically non-significant; *n* = 3.

Nrf2 is well known as a crucial modulator of cellular redox balance through upregulation of antioxidant genes like HO-1. Given that SGME activates the Nrf2/HO-1 axis, we speculated that SGME can attenuate cellular oxidative stress in LPS-stimulated macrophages. Indeed, while LPS enhanced total ROS production in macrophages, SGME prominently suppressed LPS-induced ROS (Fig. 4A). Similarly, SGME decreased the formation of mitochondrial ROS (mtROS) in LPS-activated macrophages (Fig. 4B). To verify the contribution of the Nrf2/HO-1 axis to the modulation of ROS generation by SGME, we blocked this signaling pathway with pharmacological inhibitors of Nrf2 and HO-1 and then reassessed the effects of SGME on ROS production. As shown in Fig. 4C, SGME-mediated suppression of ROS production was abrogated by either ML385 (an Nrf2 inhibitor) or SnPP (an HO-1 inhibitor) at the indicated concentrations which did not affect cell viability (Supplementary Fig. S3). Furthermore, we knocked down the Nrf2 gene using a specific siRNA (Fig. 4D) and observed that SGME reduced LPS-induced production of total ROS in naïve macrophages but not in Nrf2-deficient macrophages (Fig. 4E). Together, these findings imply that activation of the Nrf2/HO-1 axis results in alleviation of inflammation-associated ROS generation in macrophages.

### Activation of Nrf2/HO-1 signaling pathway mediates anti-inflammatory actions of SGME in macrophages

**Fig. 5:**
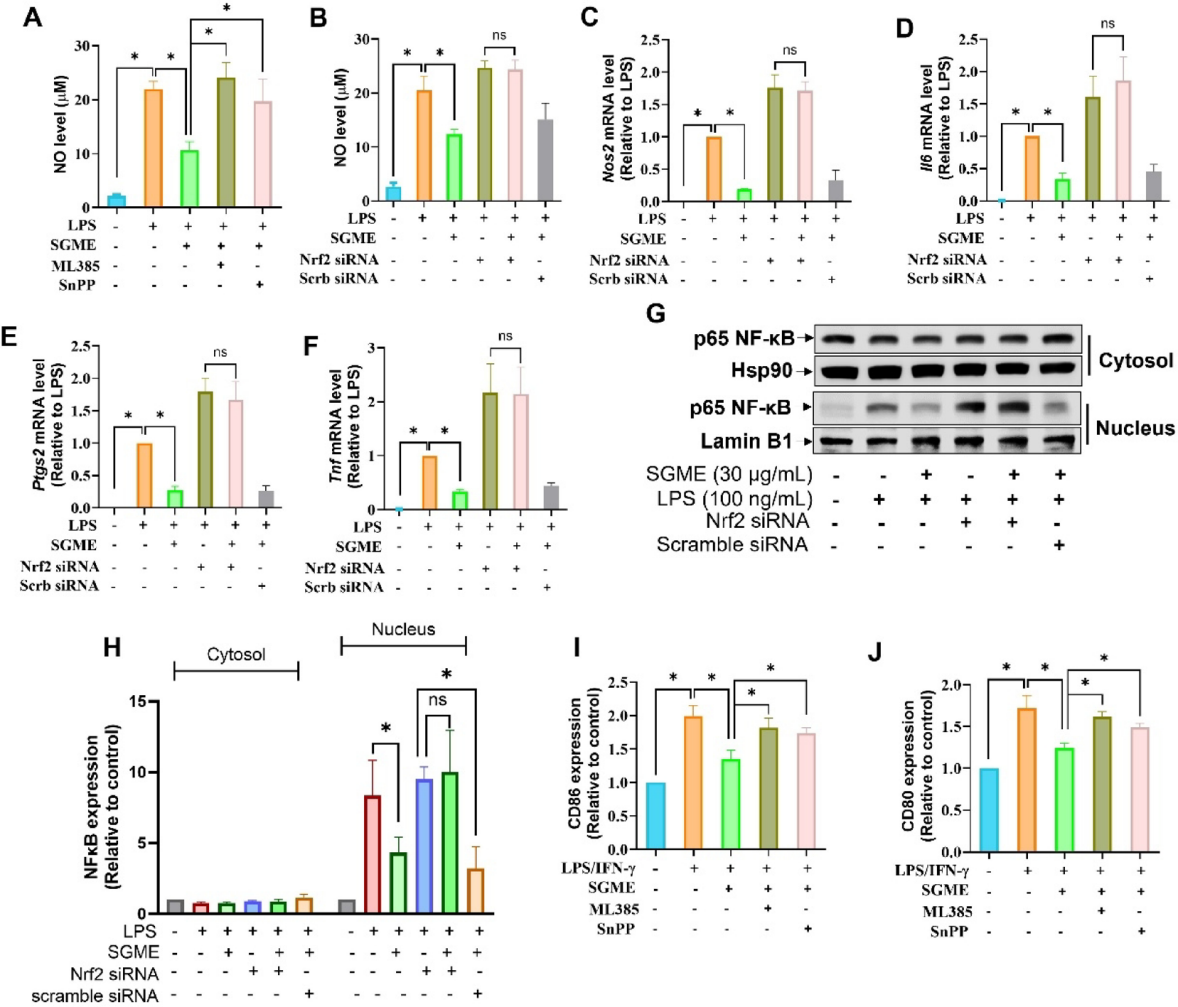
Roles of Nrf2/HO-1 in modulation of inflammatory responses by SGME. (**A**) Raw 264.7 cells were sequentially treated with ML385 (10 µM) or SnPP (10 µM) (pharmacological inhibitors of Nrf2 and HO-1, respectively) for 1 h, SGME (30 µg/mL) for 3 h, and LPS (100 ng/mL) for 24 h. Nitrite/nitrate levels were measured in culture medium. (**B**–**H)** Cells were transfected with an siRNA targeting Nrf2 (20 nM) or a scramble siRNA for 24 h. Then, cells were treated with SGME (30 µg/mL) for 3 h, followed by LPS (100 ng/mL) for 24 h (**B**), 6 h (**C**–**F**), or 1 h (**G**–**H**). (**B**) NO production was determined by measuring nitrite/nitrate release into culture medium. (**C**–**F**) The mRNA levels of genes encoding iNOS (**C**), IL-6 (**D**), COX-2 (**E**), and TNF-α (**F**) were measured using RT-qPCR. (**G **and** H**) The expression of p65 NF-κB in cytoplasmic and nuclear fractions was examined by western blot. (**I **and** J**) BMDMs were treated with ML385 (10 µM) or SnPP (10 µM) for 1 h and then with SGME (30 µg/mL) for an additional 3 h. BMDMs were next incubated with LPS/IFN-γ (100/10 ng/mL) for 24 h to induce M1 polarization. The expression of CD86 (**I**) and CD80 (**J**) on the cell surface was analyzed using flow cytometry. * denotes *p* < 0.05; ns: statistically non-significant; *n* = 3.

Having demonstrated that SGME modulates the Nrf2/HO-1/ROS signaling pathway, we next sought to verify the implication of this pathway in the anti-inflammatory actions of SGME. By measuring the release of NO metabolites into culture medium, we found that ML385 and SnPP, which are inhibitors of Nrf2 and HO-1, respectively, impeded the suppressive effect of SGME on NO production from LPS-primed macrophages (Fig. 5A). Likewise, SGME could efficiently reduce LPS-stimulated NO secretion; however, this effect was not observed in Nrf2-deficient macrophages (Fig. 5B). Also, while SGME prevented pro-inflammatory genes, including *Nos2*,* Il6*,* Ptgs2*, and *Tnf*, from being upregulated by LPS, it failed to suppress these genes in cells with Nrf2 knockdown (Fig. 5C–F). Additionally, SGME inhibited the translocation of NF-κB into the nuclear compartment in non-transfected or scrambled-siRNA transfected cells, whereas NF-κB nuclear level was unaffected by SGME in Nrf2-siRNA transfected macrophages (Fig. 5G and H). To confirm whether the Nrf2/HO-1 axis is involved in the M1 differentiation of primary macrophages, we pretreated BMDMs with pharmacological inhibitors of Nrf2 and HO-1 before re-examining the effects of SGME on M1 polarization of BMDMs. As shown in Fig. 5I and J, SGME suppressed the expression of M1 markers, including CD86 and CD80, in LPS/IFN-γ stimulated BMDMs. In contrast, the suppressive effects of SGME on these M1 markers were significantly abrogated by ML385 and SnPP, indicating that Nrf2/HO-1 is required for the inhibitory effect of SGME on M1 polarization of macrophages. Collectively, the anti-inflammatory effects of SGME are mediated, at least in part, through activation of the Nrf2/HO-1 signaling pathway.

### SGME ameliorates LPS-induced acute lung inflammation in mice

**Fig. 6:**
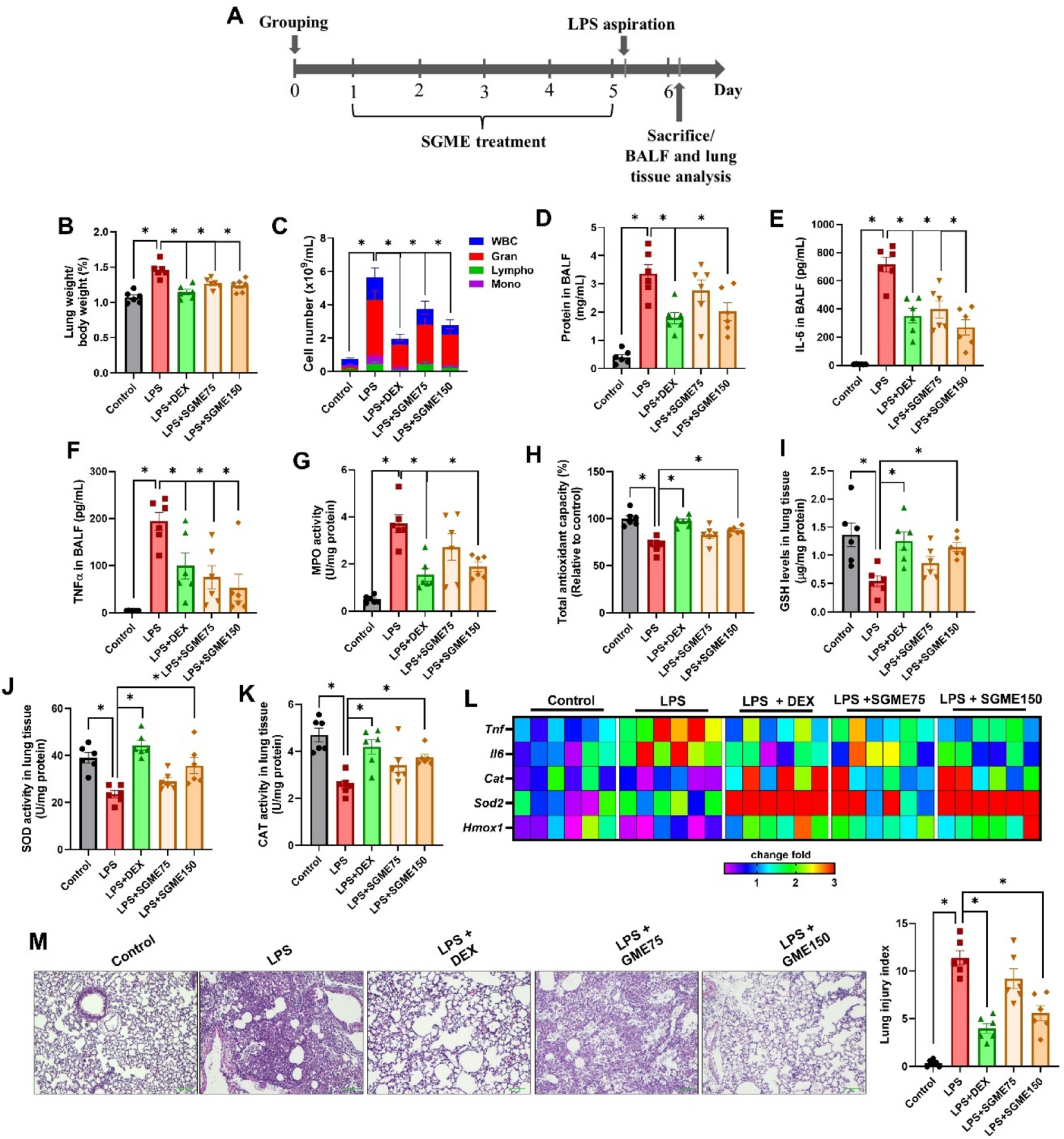
Effects of SGME against LPS-induced acute lung inflammation in mice. (**A**) A schematic illustration of experimental design. Mice were treated with SGME at doses of 75 and 150 mg/kg or dexamethasone (1 mg/kg). Then, pulmonary inflammation was induced by administration of LPS (250 µg/mouse) via oropharyngeal inhalation. After 24 h of LPS inhalation, bronchoalveolar lavage fluid (BALF) and lung tissues were collected for further analyses. (**B**) The lung-to-body weight ratio. (**C**–**F**) The number of total white blood cells (WBC) along with granulocytes (Gran), lymphocytes (Lympho), and monocytes (Mono), total protein concentration, IL-6 and TNF-α levels were determined in BALF. (**G**) Myeloperoxidase (MPO) activity was measured in lung tissue homogenates. (**H**) The total antioxidant capacity of lung tissue homogenates was examined using the ABTS^●^ free radical scavenging assay. (**I**–**K**) The levels of GSH, CAT and SOD activity were measured in lung tissue homogenates. (**L**) Total RNA was extracted from lung tissues and used to examine mRNA expression of genes encoding TNF-α (*Tnf*), IL-6 (*Il6*), catalase (*Cat*), SOD (*Sod2*), and HO-1 (*Hmox1*) by RT-qPCR analysis. The heat map presented the relative gene expression compared to the control group. GAPDH was used as a reference gene. (**M**) Representative images of H&E staining of lung tissues from each group were shown along with quantification of lung injury based on neutrophil infiltration, interstitial inflammation, edema, and congestion as described in Methods. * denotes *p* < 0.05; *n* = 6.

The in vitro anti-inflammatory effects of SGME were validated using a mouse model of LPS-induced acute lung injury (Fig. 6A). Based on preliminary dose-response experiments, mice were treated with SGME at two oral doses: 75 and 150 mg/kg. LPS inhalation resulted in an increase in the ratio of lung-to-body weight, but SGME significantly restored this ratio (Fig. 6B). LPS-induced lung inflammation was evidenced by lung infiltration of white blood cells (WBC), leading to enhanced secretion of pro-inflammatory cytokines (Fig. 6C–F). As expected, treatment with SGME prominently reduced the number of WBC, including granulocytes, lymphocytes, and monocytes (Fig. 6C), the total protein concentration (Fig. 6D), IL-6 (Fig. 6E) and TNF-α (Fig. 6F) levels in bronchoalveolar lavage fluid (BALF) in a dose-dependent manner. Furthermore, lung myeloperoxidase (MPO) activity, a marker of neutrophil infiltration, was decreased by SGME at 150 mg/kg (Fig. 6G), indicating that SGME inhibits neutrophil infiltration into lung tissues. Given our previous findings that SGME inhibits LPS-promoted ROS production, we examined markers reflecting the antioxidant capacity of lung tissues in LPS-inhaled mice. Total antioxidant capacity, which was estimated using the ABTS^●^ scavenging activity assay, decreased in lung tissues derived from LPS-treated mice. By contrast, oral administration of SGME improved total antioxidant capacity in lung tissues (Fig. 6H). Likewise, while LPS impaired GSH levels as well as SOD and CAT activity in lung tissues, SGME significantly abrogated these detrimental effects of LPS on the antioxidant system of lung tissues (Fig. 6I–K). The RT-qPCR analysis further confirmed that SGME reduced the expression of pro-inflammatory genes, including IL-6 and TNF-α, but increased the expression of Nrf2-associated genes, including HO-1, CAT, and SOD (Fig. 6L and Supplementary Fig. S4). Consistent with changes in biochemical markers, H&E staining images showed that LPS inhalation induced abnormal alterations in lung tissue architecture, including severe infiltration of inflammatory cells, increased septal thickness, and interstitial and alveolar edema and congestion (Fig. 6M). By contrast, SGME alleviated the lung injury index in LPS-inhaled mice. Notably, the beneficial effects of SGME against LPS-induced lung inflammation were comparable with those of dexamethasone (1 mg/kg). Taken together, the in vivo study demonstrated that oral administration of SGME effectively attenuated inflammatory responses and oxidative stress in lung tissues of LPS-treated mice.

## Discussion

The Nrf2/HO-1 pathway is a well-recognized target for regulating inflammatory responses and oxidative stress, and numerous plant-derived compounds exert anti-inflammatory effects through this mechanism^31^. *G. montanum*, a medicinal plant rich in stilbenes, such as resveratrol, isorhapontigenin, gnetol, and gnetulin, has traditionally been used for the treatment of inflammatory disorders; however, its mechanistic effects on inflammation have remained unclear. In the present study, SGME activated the Nrf2/HO-1 axis in macrophages, leading to suppression of NF-κB signaling, reduced production of pro-inflammatory mediators, attenuation of M1 macrophage polarization, and decreased oxidative stress. Together, these findings support a mechanistic framework in which SGME activates Nrf2/HO-1 to limit inflammation-associated ROS generation and thereby dampen NF-κB–dependent inflammatory outputs. Consistent with these in vitro observations, SGME also attenuated LPS-induced acute lung inflammation in mice under a pretreatment paradigm, providing an initial basis for further investigation of SGME in inflammation-associated lung disease.

To characterize the anti-inflammatory actions of SGME, we used Raw 264.7 macrophages for mechanistic profiling and primary BMDMs to validate key findings in a non-transformed system. In Raw 264.7 macrophages, SGME suppressed LPS-induced NF-κB nuclear translocation and reduced the expression of NF-κB-dependent pro-inflammatory genes, including *Nos2*, *Ptgs2*, *Il6*, and *Tnf*. This transcriptional suppression was accompanied by a significant decrease in the secretion of NO, PGE₂, IL-6, and TNF-α, key mediators contributing to the inflammatory microenvironment. While Raw 264.7 cells provide a robust and highly reproducible system to define SGME’s core anti-inflammatory and redox-modulating actions and to enable detailed mechanistic readouts, their transformed nature may not fully recapitulate primary macrophage polarization^32^. Therefore, we next validated SGME in primary BMDMs, which predominantly exhibit an M0 phenotype after differentiation and are widely used for M1 polarization studies^33^. Macrophages orchestrate inflammatory responses by adopting a pro-inflammatory M1 phenotype under inflammatory cues^34^, typically characterized by increased CD80/CD86 expression and elevated production of NO and pro-inflammatory cytokines^35^. Notably, SGME suppressed the induction of M1 markers and NO production in BMDMs stimulated with LPS and IFN-γ. IFN-γ primes macrophages through the JAK/STAT1–IRF1 axis, enhancing iNOS-related machinery and co-stimulatory/antigen-presentation programs, whereas LPS provides a strong TLR4/NF-κB–driven inflammatory transcriptional signal^33,36^. Accordingly, LPS/IFN-γ co-stimulation is a well-established in vitro model of classical (M1) polarization; therefore, our findings indicate that SGME restrains M1 programming under these polarizing conditions.

To clarify the mechanism, we found that brief exposure of macrophages to SGME promoted nuclear translocation of Nrf2, leading to the upregulation of Nrf2-regulated genes, including *Hmox1*,* Sod2*, and *Cat*. Notably, the anti-inflammatory effects of SGME were largely abolished in Nrf2-deficient macrophages, emphasizing the central role of Nrf2 activation in mediating these responses. Previous studies have demonstrated that Nrf2 modulates inflammation both directly and indirectly. For instance, in LPS-activated macrophages, Nrf2 can repress pro-inflammatory cytokine transcription by interacting with target promoters and limiting RNA polymerase II recruitment, independently of its canonical binding motif and redox regulation^37^. Moreover, Nrf2 loss enhances, whereas Nrf2 overexpression suppresses, NF-κB activation^38^. Together, these findings indicate that Nrf2 exerts anti-inflammatory effects by downregulating NF-κB–dependent gene expression, thereby inhibiting M1 polarization and inflammation activation in vitro and in vivo. In line with these established functions of Nrf2, our findings suggest that SGME modulates macrophage-mediated inflammation by inhibiting NF-κB activation, suppressing the upregulation of pro-inflammatory mediators, and preventing M1 polarization. These effects are mediated, at least in part, through Nrf2 activation.

In addition to suppressing NF-κB activation and pro-inflammatory gene expression, Nrf2 exerts anti-inflammatory effects by regulating target genes and maintaining cellular redox homeostasis^39^. In our study, SGME markedly upregulated HO-1 in macrophages, and pharmacological inhibition of HO-1 weakened the anti-inflammatory effects of SGME, supporting HO-1 induction as a functional component of SGME activity. Notably, HO-1, a key Nrf2 downstream effector, contributes to Nrf2–NF-κB crosstalk; its metabolites (e.g., biliverdin and carbon monoxide) can inhibit NF-κB nuclear translocation and transcriptional activity, potentially via interaction with the p65 subunit^40,41^. Moreover, HO-1, together with other Nrf2-regulated antioxidant enzymes (CAT, SOD, and NQO1), helps relieve oxidative stress, a key driver of inflammation-associated tissue damage^42^. In inflammatory macrophages, ROS is not only generated downstream of pro-inflammatory signaling but can also act upstream to amplify NF-κB activation and sustain transcription of inflammatory mediators, creating a ROS–NF-κB feed-forward amplification loop^7^. Consistent with an Nrf2/HO-1–dependent redox mechanism, SGME reduced inflammation-associated ROS generation, including mtROS, and this ROS-lowering effect was attenuated when Nrf2/HO-1 signaling was pharmacologically inhibited or when Nrf2 was knocked down. Given the ROS–NF-κB feed-forward loop, the concurrent loss of SGME-mediated ROS suppression and NF-κB inhibition in Nrf2-deficient settings suggests that redox control is a key mechanism by which SGME disrupts inflammatory amplification.

Using a well-established LPS-induced acute lung inflammation model, we showed that SGME attenuated pulmonary inflammation, as reflected by reduced inflammatory cell infiltration and lower levels of pro-inflammatory cytokines in bronchoalveolar lavage fluid. Furthermore, SGME also alleviated pulmonary oxidative stress, accompanied by upregulation of key Nrf2 target genes, including *Hmox1*,* Cat*, and *Sod2*. These findings support the concept that the anti-inflammatory and antioxidative effects of SGME are mediated, at least in part, through activation of the Nrf2/HO-1 pathway and modulation of redox balance. ALI is a severe respiratory disorder characterized by increased alveolar permeability, pulmonary edema, immune cell infiltration, excessive inflammation, and epithelial damage^43^. Uncontrolled inflammatory responses substantially contribute to the high morbidity and mortality associated with ALI^44^, and although corticosteroids have shown benefit in some respiratory conditions, their broad immunosuppressive effects restrict clinical applicability^45^. Therefore, there is an urgent need for more selective anti-inflammatory therapies for ALI. In this context, the attenuation of lung inflammation by SGME in the LPS-induced model supports further investigation of its effects in inflammatory lung disease. However, because the in vivo experiments were performed in a pretreatment setting, the present results should be interpreted as preliminary evidence in an experimental acute inflammatory model rather than as proof of therapeutic efficacy in established lung disease. This model nevertheless provides a biologically relevant setting in which alveolar macrophages are among the earliest responders to inhaled LPS and serve as key sources of cytokines/chemokines that drive neutrophil recruitment, MPO activity, and tissue injury; thus, the macrophage-centered mechanism identified in vitro offers a plausible link to the in vivo lung phenotype^46^.

Although SGME contains a chemically complex mixture of constituents, the present study was not designed to identify the specific bioactive components responsible for the observed effects. Therefore, the overall phenotype may reflect the action of different active constituents or interactions among multiple compounds present in the extract. SGME was rich in polyphenols, which are widely recognized for their anti-inflammatory and antioxidant properties^47^. Among the identified compounds, isorhapontigenin was particularly abundant. Notably, a recent study demonstrated that this stilbene protected mice against LPS-induced ALI by inhibiting NF-κB and activating Nrf2^24^. Another bioactive stilbene detected in SGME, resveratrol, has been shown to attenuate LPS-induced ALI through Nrf2/HO-1 upregulation in lung tissue^48^. Both isorhapontigenin and resveratrol reduce oxidative stress by enhancing the expression of antioxidant enzymes such as SOD and CAT^49,50^. In this regard, the pattern observed with SGME, including induction of Nrf2-associated antioxidant genes, reduction of ROS, and suppression of inflammatory mediators, is essentially similar to that reported for these known Nrf2 activators in inflammatory lung models. At the same time, the present data do not allow us to determine whether the activity of SGME is driven primarily by a single major stilbene or by additive or synergistic interactions among multiple constituents. Further studies using bioactivity-guided fractionation, purified compounds, and reconstitution approaches will be needed to elucidate the relative contributions of individual constituents and multi-component interactions.

While the present study evaluated SGME in an acute inflammation setting using established inflammatory readouts and M1 macrophage polarization markers, it remains unclear how SGME modulates inflammatory responses under chronic inflammatory conditions and whether its effects on M2 polarization and inflammation resolution contribute to its overall anti-inflammatory properties. These gaps highlight the need to examine SGME in animal models of chronic inflammation to better define its inflammation-modulatory profile and strengthen the translational relevance of our findings. In addition, although *G. montanum* extract is rich in stilbene derivatives, key pharmacological data are currently unavailable, including the oral bioavailability and pharmacokinetics of major constituents and the relationship between pharmacokinetics and pharmacodynamics (PK/PD). Addressing these aspects in future studies will be essential for interpreting exposure-response relationships and for more rigorously assessing the therapeutic potential of SGME in inflammation-related diseases.

In summary, this study provides novel evidence that SGME exerts significant anti-inflammatory effects through modulation of Nrf2-driven signaling pathways in a model of lung inflammation. By promoting nuclear translocation of Nrf2, SGME enhances the expression of key antioxidant and cytoprotective genes, including *Hmox1*, *Cat*, and *Sod2*. Through Nrf2 activation and its downstream targets, SGME suppresses inflammatory responses by directly inhibiting NF-κB–dependent gene transcription, reducing pro-inflammatory mediator secretion and preventing M1 macrophage polarization. In parallel, SGME-dependent Nrf2/HO-1 activation contributes to reduced inflammation-associated ROS generation, which is expected to disrupt the pathological amplification cycle between oxidative stress and inflammatory transcriptional programs. In the LPS-induced acute lung inflammation model, these mechanisms were associated with attenuation of inflammatory responses in vivo. Overall, our findings support SGME as a biologically plausible source of Nrf2-modulating anti-inflammatory activity and provide a foundation for future studies aimed at identifying active constituents and evaluating efficacy in more relevant therapeutic settings.

## Materials and methods

### Chemicals and reagents

Reagents for cell culture (culture medium, fetal bovine serum, bovine serum albumin, antibiotics and other culture supplements) were acquired from PAN-Biotech GmbH (Aidenbach, Germany); Lipopolysaccharide (LPS) (#LPS25), 5,5’-dithiobis-(2-nitrobenzoic acid) (#D8130); thiobarbituric acid (#T5500), pyrogallol (#16040), 2,2′-azino-bis(3-ethylbenzothiazoline-6-sulfonic acid) diammonium salt (ABTS) (#A1888), and solvents for extraction and chemical analysis of plant extract were provided by Sigma-Aldrich (St. Louis, MO, USA). ML385 (#HY-100523), Tin protoporphyrin IX dichloride (SnPP) (#HY-101194), NG-Nitroarginine methyl ester hydrochloride (L-NAME) (#HY-18729 A), dexamethasone (#HY-14648), N-acetylcysteine (#HY-B0215), and Mitoquinone mesylate (mitoQ) (#HY-100116 A) were purchased from MedChemExpress (Monmouth Junction, NJ, USA). IFN-γ (#575304) and fluorescence-conjugated primary antibodies for flow cytometry, including anti-F4/80 (PE) (#123110), anti-CD11b (APC) (#101212), anti-CD80 (FITC) (#104705), and anti-CD86 (APC/Cy7) (#105029), were procured from BioLegend (San Diego, CA, USA). Primary antibodies against p65 NF-κB (#sc-372) and phospho-p65 (#sc-136548) were purchased from Santa Cruz Biotechnology (Dallas, Texas, USA); antibodies against Nrf2 (#12721) and HO-1 (#43966) were obtained from Cell Signaling Technology (Beverly, MA, USA); antibodies against Lamin B1 (#MAB8525) and Hsp90 (#MAB3286) were purchased from R&D Systems (Minneapolis, MN, USA); antibody against β-actin (#MA5-15739) and HRP-conjugated secondary antibodies against mouse (#31430) and rabbit (#31460) IgG were procured from Thermo Scientific (Waltham, MA, USA). Alexa Fluor 488 conjugated anti-rabbit secondary antibody (#ab150077) was acquired from Abcam (Cambridge, MA, USA).

### Preparation and chemical characterization of plant extract

Stems and branches of *Gnetum montanum* Markgr. were collected at Muong Nhe (Dien Bien, Vietnam), during the blooming period in April 2022. The collection was carried out in non-protected areas where sampling of this species is legally permitted and was conducted in compliance with national and international guidelines, including the IUCN Policy Statement on Research Involving Species at Risk of Extinction and the Convention on International Trade in Endangered Species of Wild Fauna and Flora (CITES). Permissions for field sampling and specimen collection were obtained from the appropriate local authorities in accordance with Vietnamese legislation. The species was authenticated by Dr. The-Bach Tran at the Institute of Ecology and Biological Resources, Vietnam Academy of Science and Technology (IEBR-VAST). A voucher specimen (DL2) has been deposited at the same institute.

The raw materials were processed and extracted following a previously described protocol^51^. Briefly, the collected plant parts were washed, dried at 50 °C, and ground into a fine powder. The powder (3 × 2000 g) was extracted with 95% ethanol by reflux (three times for 3 h each), then concentrated and oven-dried, yielding 1008 g of a thick brown extract (13.2% yield with a 21.5% moisture loss). A single batch of plant extract was prepared and used consistently across all in vitro and in vivo experiments. The total phenolic content was determined to be 25.36 mg GAE/g using the Folin-Ciocalteu method^51^.

Stilbene compounds in the stilbene-rich *G. montanum* extract (SGME) were analyzed using a Shimadzu HPLC system equipped with an SPD-M20A DAD detector (320 nm) and a C18 column (250 × 4.6 mm; 5 μm, Agilent). The mobile phase consisted of a gradient of acetonitrile (solvent A) and 0.01% formic acid in water (solvent B) at a flow rate of 0.8 mL/min, according to the following program: 0–10 min (5% A), 10–70 min (5–30% A), 70–90 min (30–95% A), and 90–95 min (95% A).

**Fig. 7:**
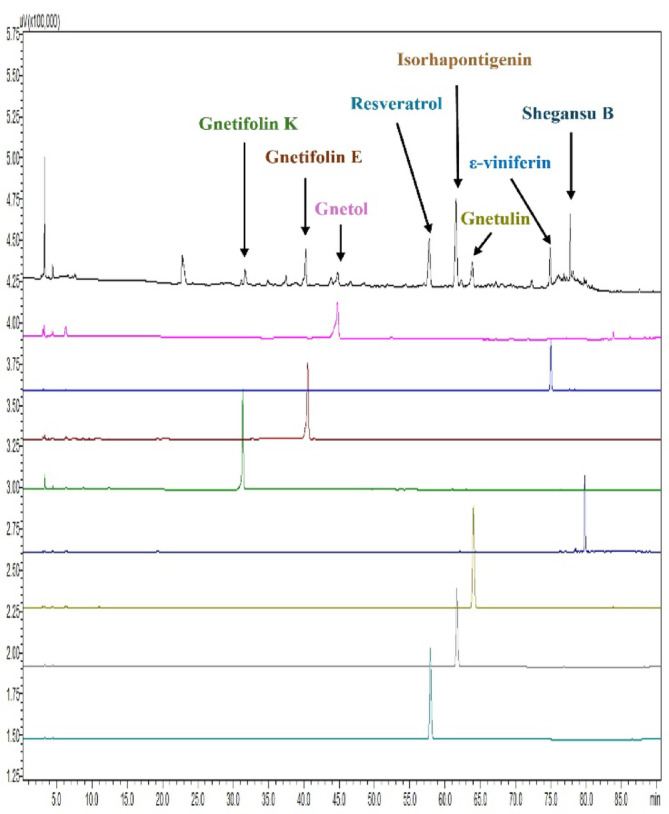
HPLC chromatogram of crude ethanol extract and chemical compounds from *Gnetum montanum*. Line 1: SGME, Line 2: Gnetol, Line 3: *ε*-viniferin, Line 4: Gnetifolin E, Line 5: Gnetifolin K, Line 6: Shegansu B, Line 7: Gnetulin, Line 8: Isorhapontigenin, Line 9: Resveratrol.

Compounds were identified by comparing their retention times (tR) and spectroscopic data (¹H- and ¹³C-NMR) with those of authentic standards. As shown in Fig. 7, the stilbene derivatives identified in SGME included gnetifolin K (line 5; tR = 31.2 min), gnetifolin E (line 4; tR = 40.52 min), Gnetol (line 2; tR = 44.63 min), resveratrol (line 9; tR = 58.03 min), isorhapontigenin (line 8; tR = 61.91 min), gnetulin (line 7; tR = 64.11 min), ε-viniferin (line 3; tR = 74.90 min), and shegansu B (line 6; tR = 79.81 min). Quantitative analysis further revealed that the concentrations of resveratrol and isorhapontigenin in SGME were 0.13 ± 0.00% (w/w) and 0.56 ± 0.02% (w/w), respectively (quantification data are presented in Fig. S5).

### Cell culture and treatment

Raw 264.7 macrophages were obtained from the American Type Culture Collection (ATCC, Rockville, MD, USA). Cells were routinely cultured in high-glucose DMEM supplemented with 10% fetal bovine serum (FBS) and 1% antibiotic-antimycotic solution and maintained at 37 °C in a humidified incubator with 5% CO₂.

For anti-inflammatory assays, SGME was dissolved in DMSO and added to the culture medium. After a 3-h pretreatment, cells were stimulated with lipopolysaccharide (LPS; 100 ng/mL) for durations ranging from 30 min to 24 h, depending on the specific assay. During treatment, cells were maintained in medium containing 0.1% FBS.

### Animal handling and ethical statement

Five-week-old male Swiss mice (initial body weight 18–20 g) were procured from the National Institute of Hygiene and Epidemiology (Hanoi, Vietnam). Animals were acclimated for 5–7 days before experimental procedures. After completion of each experiment, the animals were euthanized by exposure to 100% CO₂. All animal experiments were conducted in accordance with the regulations of the Animal Center at Hanoi University of Pharmacy and in compliance with the ARRIVE guidelines. The study protocol was reviewed and approved by the Animal Ethics Committee of Hanoi University of Pharmacy AEC-HUP (approval number: 1474/QD-DHN).

### Isolation, differentiation, and polarization of bone marrow-derived macrophages (BMDMs)

Bone marrow cells were harvested from the femurs of 5–7-week-old Swiss mice. After red blood cell removal using RBC Lysis Buffer (#420301, BioLegend), the cells were cultured in complete DMEM supplemented with 20 ng/mL recombinant GM-CSF (#576306, BioLegend) to induce macrophage differentiation. After 7 days of incubation, differentiated macrophages were harvested for subsequent experiments.

Following differentiation, BMDMs were pretreated with SGME for 3 h, then cultured in M1 polarization medium (DMEM containing IFN-γ (20 ng/mL) and LPS (100 ng/mL)) for an additional 24 h. Cells were then collected and stained with a cocktail of fluorescence-conjugated antibodies targeting mouse F4/80 (PE), CD11b (APC), CD80 (FITC), and CD86 (APC/Cy7) for 15 min on ice. Flow cytometry analysis was performed using a BD FACSVerse system (BD Biosciences, San Jose, CA, USA).

### Cell viability assay

Cells were seeded in 96-well plates at a density of 4 × 10⁴ cells/well. Following treatment with various concentrations of SGME, cells were incubated with 0.5 mg/mL 3-(4,5-Dimethylthiazol-2-yl)-2,5-Diphenyltetrazolium Bromide (MTT reagent; #M6494, Thermo Scientific) for 2 h. Formazan crystals formed were dissolved in DMSO, and absorbance was measured at 570 nm using a microplate reader (Varioskan LUX, Thermo Scientific).

### Quantification of pro-inflammatory mediators

After SGME treatment, cells were stimulated with LPS for 24 h. The culture medium was collected and stored at -80 °C for subsequent analyses, including nitric oxide (NO), IL-6, PGE2, and TNF-α assays. NO levels were quantified using a modified Griess method with the Griess Reagent System (#G2930, Promega). Concentrations of IL-6, TNF-α, and PGE2 were determined by ELISA using commercial kits from BioLegend (#431304 for IL-6 and #430904 for TNF-α) and Abcam (#ab316263 for PGE2), following the manufacturers’ protocols.

### Measurement of total and mitochondrial reactive oxygen species (ROS)

Total and mitochondrial ROS levels were assessed after 24 h of LPS stimulation as described previously^52^. Cells were incubated with fluorescent indicators specific for total ROS (CM-H₂DCFDA; #C6827, Thermo Scientific) and mitochondrial ROS (MitoSOX Red; #M36008, Thermo Scientific) for 30 min at 37 °C. After incubation, cells were harvested by scraping, and mean fluorescence intensity (MFI) was measured by flow cytometry. Data were expressed as relative MFI values compared to control.

### Gene silencing with small interfering RNA (siRNA)

Cells were cultured to approximately 60–70% confluence before transfection with either an siRNA targeting Nrf2 or a scramble control siRNA, using Lipofectamine RNAiMAX Transfection Reagent (#13778100, Thermo Scientific) according to the manufacturer’s instructions. After 24 h, cells were harvested either for assessment of transfection efficiency or for subsequent experiments. The siRNA primer sequences used in this study are listed in Table S1.

### Reverse transcription and quantitative polymerase chain reaction (RT-qPCR)

Following treatment, cells were lysed with TRIzol Reagent (#15596026; Thermo Scientific). Total RNA was extracted using chloroform, precipitated with 2-propanol, and washed with 75% ethanol. Purified RNA (1 µg) was reverse transcribed into complementary DNA (cDNA) using the Reverse Transcription System (#A3500; Promega) according to the manufacturer’s instructions. Quantitative PCR (qPCR) was subsequently performed using SYBR Green Master Mix (#4309155; Thermo Scientific) in a 96-well plate format on a 7500 Real-Time PCR System (Thermo Scientific). Reaction conditions were established based on previously described protocols^53^. Primer sequences used for RT-qPCR assays are listed in Table S1.

### Gel electrophoresis and western blot analysis

Cells were lysed using either RIPA lysis buffer (#89900; Thermo Scientific) to obtain total protein or a subcellular protein fractionation kit (#78840; Thermo Scientific) to separate cytoplasmic and nuclear proteins. Protein concentrations were determined using the BCA assay (#A55860, Thermo Scientific). Equal amounts of protein (20 µg) were separated by sodium dodecyl sulfate-polyacrylamide gel electrophoresis (SDS-PAGE) and transferred onto PVDF membranes. Membranes were blocked with 3% bovine serum albumin (BSA) for 60 min at room temperature, then incubated overnight at 4 °C with primary antibodies against the proteins of interest, followed by incubation with HRP-conjugated secondary antibodies (60 min at room temperature). Immunoreactive bands were visualized using SuperSignal West Pico PLUS Chemiluminescent Substrate (#34580; Thermo Scientific) and detected with a gel imaging system (Fujifilm, Tokyo, Japan). Band intensities were quantified using ImageJ software based on three independent experiments.

### Immunocytochemistry (ICC)

Cells were cultured in an 8-well glass chamber slide. After treatment, cells were fixed with 4% paraformaldehyde for 20 min at room temperature, followed by permeabilization with 0.2% Triton X-100 in phosphate-buffered saline (PBS). Cells were then blocked with 5% normal goat serum for 1 h and sequentially incubated with primary antibodies against Nrf2 or NF-κB (overnight at 4 °C), followed by incubation with Alexa Fluor 488-conjugated secondary antibodies for 90 min at room temperature. After removing the chamber walls, the slides were mounted with a DAPI-containing mounting medium (#ab104139; Abcam). Fluorescence images were captured using a confocal microscope (Nikon, Tokyo, Japan).

#### *Establishment of a mouse model of lipopolysaccharide (LPS) induced lung inflammation*,* treatment*,* and in vivo anti-inflammatory evaluation*

Acute lung inflammation was induced in eight-week-old male Swiss mice by oropharyngeal administration of LPS. The SGME doses (75 and 150 mg/kg/day) were selected based on a preliminary pilot dose-finding study, in which a human-equivalent dose framework was used to define an initial dose range, and doses were subsequently adjusted according to the observed biological responses to identify two effective levels for the main experiment. Mice were randomly divided into five groups (*n* = 6 per group): normal control, LPS control, positive control, and two SGME treatment groups. The treatment groups received SGME at doses of 75 or 150 mg/kg/day, while the positive control group received dexamethasone at 1 mg/kg/day. The normal and LPS control groups were administered normal saline at an equivalent volume. All treatments were given via oral gavage for five consecutive days. On day 5, one hour after the final treatment, mice were anesthetized with isoflurane and 50 µL of LPS (5 mg/mL) was administered via oropharyngeal inhalation into the tracheal bronchus. After 24 h, mice were euthanized; the trachea was exposed, and bronchoalveolar lavage fluid (BALF) was collected by flushing the lungs with 1 mL PBS/EDTA buffer. A portion of the BALF was used to count the total number of leukocytes using a URIT-3000Vet Plus Analyzer (URIT Medical Electronic, Guangxi, China). The remaining BALF was then centrifuged to obtain the supernatant for downstream assays. Lung tissues were subsequently dissected, weighed, and processed for further analyses.

#### Measurement of parameters in bronchoalveolar lavage fluid (BALF)

Levels of TNF-α and IL-6 were quantified by ELISA using commercial kits (#431304 and #430904, respectively), following the manufacturer’s instructions. Protein content was determined by the BCA method.

### Measurement of antioxidant markers in lung tissues

Lung tissues were homogenized in ice-cold PBS (1:10 w/v) using a homogenizer (DAIHAN Scientific, Gangwon-do, Korea). After centrifugation at 10,000 rpm for 20 min, the supernatants were collected for the measurement of myeloperoxidase (MPO) activity, malondialdehyde (MDA), reduced glutathione (GSH), superoxide dismutase (SOD), catalase (CAT), and ABTS radical scavenging activity. All parameters were normalized to the total protein content measured by the BCA method.

MPO activity was assessed as previously described and normalized to the total protein concentration in lung homogenates^54^.

MDA levels were determined using the thiobarbituric acid reactive substances (TBARS) assay. Homogenates were mixed with thiobarbituric acid (0.5% in 50% acetic acid) and heated at 95 °C for 60 min. Absorbance was measured at 532 nm, and MDA concentrations were calculated based on a standard curve prepared with tetramethoxypropane.

GSH content was assessed using the Ellman reagent. Lung homogenates were mixed with 5,5’-dithiobis-(2-nitrobenzoic acid) (10 mM in phosphate buffer), and absorbance was read at 412 nm.

CAT activity was measured following the method of Hadwan and Abed^55^, based on the decomposition of H₂O₂. The reaction mixture contained 5 µL of lung homogenate and 50 µL of 50 mM H₂O₂. After a 3-minute incubation at 37 °C, the reaction was stopped with 200 µL of 32.4 mM ammonium molybdate, and absorbance was measured at 374 nm.

SOD activity was determined using the pyrogallol autoxidation method^56^. Briefly, 5 µL of lung homogenate was incubated with 20 µL of pyrogallol (3.25 mM in 0.1 mM HCl) and 160 µL of phosphate buffer (pH 8.5). Absorbance was recorded at 5 and 10 min post-incubation. SOD activity was calculated using the formula: SOD activity (kU/L) = 0.02×[(100–100×𝛥OD_sample_/ 𝛥OD_control_)]/V, where 𝛥OD = OD_10 min_ – OD_5 min_ in the presence (sample) or absence (control) of tissue homogenates.

Total antioxidant capacity was evaluated by measuring ABTS· radical scavenging activity. The reaction mixture contained 5 µL of lung homogenate and 195 µL of ABTS· solution (7 mM ABTS and 2.45 mM potassium persulfate). After a 2-minute incubation at room temperature, absorbance was measured at 734 nm. Radical scavenging activity was calculated based on the reduction in absorbance relative to the negative control.

#### Gene expression analysis

A portion of fresh lung tissue was homogenized in TRIzol reagent, and total RNA was extracted following the same procedure used for cell samples. The mRNA levels of *Tnf*, *Il6*, *Cat*, *Sod2*, and *Hmox1* were determined by RT-qPCR as described above.

#### Hematoxylin and eosin (H&E) staining

The remaining lung tissues were fixed in 4% paraformaldehyde for 24 h at 4 °C. Tissues were embedded in paraffin, and Sect. (4 μm thickness) were prepared using a microtome. Histopathological changes were observed after staining with hematoxylin and eosin (H&E) using a commercial kit (#C0105S; Beyotime Biotechnology, Shanghai, China) following the manufacturer’s instructions. Images were captured using a light microscope (Nikon, Tokyo, Japan). Lung histopathology was evaluated using a semi-quantitative lung injury scoring system adapted from a previously published protocol^57^. Tissue damage was scored across four criteria, including neutrophil infiltration, interstitial inflammation, edema, and congestion, on a 0–4 scale (0: none/normal; 1: involvement of ≤ 25% of the field; 2: ≤50% of the field; 3: ≤75% of the field; 4: diffuse involvement). Scoring was conducted by a pathologist blinded to group allocation. For each animal, scores from five randomly selected fields per section were averaged to generate the overall lung injury score.

### Statistical analysis

Data are presented as mean ± standard error of the mean (SEM). For in vitro experiments, values represent three independent biological replicates unless otherwise stated. For in vivo experiments, n indicates the number of animals per group as specified in the figure legends. All data points were included in the final analyses. Statistical significance among groups was evaluated using one-way analysis of variance (ANOVA) in GraphPad Prism 10.4 (San Diego, CA, USA), followed by Tukey’s post-hoc test. A p-value < 0.05 was considered statistically significant.

## Supplementary Information

Below is the link to the electronic supplementary material.

Supplementary Material 1

## Data Availability

Data is available upon request from the Authors.
