# Supplementary material for: Gnetum montanum extract attenuates lipopolysaccharide induced acute lung inflammation through Nrf2 and heme oxygenase 1 mediated redox modulation in macrophages
Source: Sci Rep. 2026 Apr 24;16:18995. doi: 10.1038/s41598-026-50370-z (PMC13276079; doi:10.1038/s41598-026-50370-z)

Journal: Scientific Reports

Stilbene-rich extract from *Gnetum montanum* Markgr. attenuates LPS-induced acute lung inflammation via Nrf2/HO-1-mediated redox modulation in macrophages

Duc-Vinh Pham[a], Hong-Linh Tran[a], Thu-Hang Nguyen[a], Hong-Nhung Bui[a], Hai-Nam Nguyen[b], Thanh Nguyen Le[c], Thi Hien Pham[d], Thuy-Duong Nguyen[a*]

[a] Department of Pharmacology, Hanoi University of Pharmacy, 13-15 Le Thanh Tong, Cua Nam, Hanoi, Vietnam

[b] Department of Medicinal Chemistry, Hanoi University of Pharmacy, 13-15 Le Thanh Tong, Cua Nam, Hanoi, Vietnam

[c] Department of Analytical Chemistry and Standardization, National Institute of Medicinal Materials, 3B Quang Trung, Cua Nam, Hanoi, Vietnam

Corresponding Author:
Thuy-Duong Nguyen, PhD

Department of Pharmacology, Hanoi University of Pharmacy, Hanoi, Vietnam
Phone: +84913343454
Email: duongnt@hup.edu.vn

Table S1. Primer sequences used in siRNA transfection and RT-qPCR experiments

**Target gene**	**Primer**	**Nucleotide sequence**
Primer sequences for RT-qPCR		
Gapdh	Forward	5’-CATCACTGCCACCCAGAAGACTG-3’
	Reverse	5’-ATGCCAGTGAGCTTCCCGTTCAG-3'
Nos2	Forward	5'-GAGACAGGGAAGTCTGAAGCAC-3'
	Reverse	5'-CCAGCAGTAGTTGCTCCTCTTC-3'
Il6	Forward	5'-TACCACTTCACAAGTCGGAGGC-3'
	Reverse	5'-CTGCAAGTGCATCATCGTTGTTC-3'
Ptgs2	Forward	5'-GCGACATACTCAAGCAGGAGCA-3'
	Reverse	5'-AGTGGTAACCGCTCAGGTGTTG-3'
Cat	Forward	5'-CGGCACATGAATGGCTATGGATC-3'
	Reverse	5'-AAGCCTTCCTGCCTCTCCAACA-3'
Sod2	Forward	5'-TAACGCGCAGATCATGCAGCTG-3'
	Reverse	5'-AGGCTGAAGAGCGACCTGAGTT-3'
Hmox1	Forward	5'- CACTCTGGAGATGACACCTGAG-3'
	Reverse	5'- GTGTTCCTCTGTCAGCATCACC-3'
Tnf	Forward	5'- GGTGCCTATGTCTCAGCCTCTT-3'
	Reverse	5'- GCCATAGAACTGATGAGAGGGAG-3'
Primer sequences for siRNA		
Nrf2	Forward	5’-CUCAGUUUCAACUGGAUGA-3’
	Reverse	5’-UCAUCCAGUUGAAACUGAG-3'
Scramble siRNA	Forward	5’-CCUACGCCACCAAUUUCGU-3’
	Reverse	5’-ACGAAAUUGGUGGCGUAGG-3'

Figure S1.

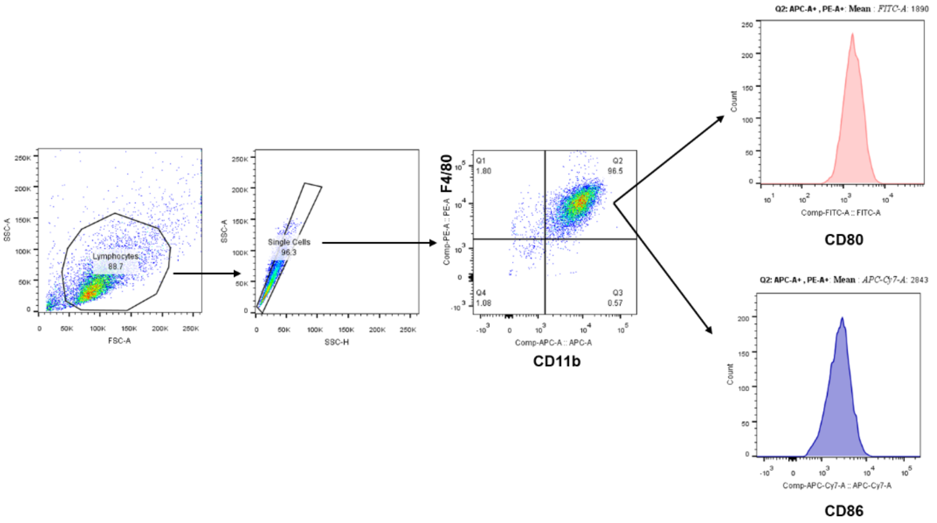

Fig. S1. Gating strategy for M1 phenotyping. Macrophages were identified as CD11b$^+$F4/80$^+$ cells, and CD80 and CD86 expression was quantified as mean fluorescence intensity (MFI).

Figure S2

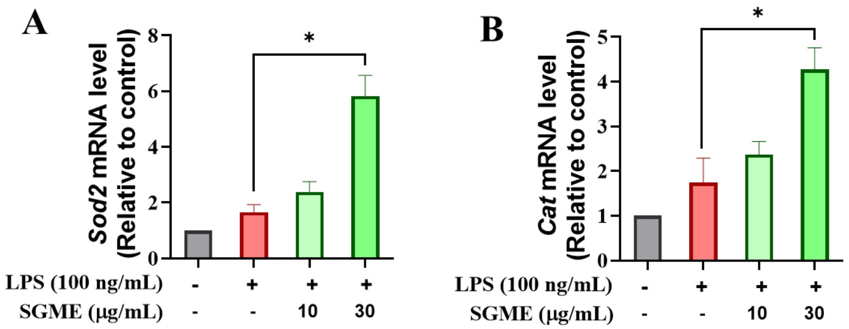

Fig. S2. Effects of SGME on the gene expression of *Sod2* and *Cat* in macrophages. (**A and B**) Raw 264.7 macrophages were pretreated with SGME for 3 h, followed by stimulation with LPS for 3 h. The mRNA levels of *Sod2* (A) and *Cat* (B) were examined by the RT-qPCR assay. * denotes $p < 0.05$; n = 3.

Figure S3.

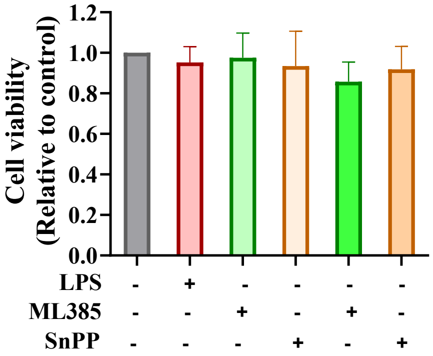

Fig. S3. Effects of ML385 and SnPP on cell viability of Raw 264.7 macrophages. Cells were treated with ML385 (10 μM) or SnPP (10 μM) for 1 h with or without stimulation with LPS (100 ng/mL) for further 24 h. Cell viability was examined by MTT assay.

Figure S4

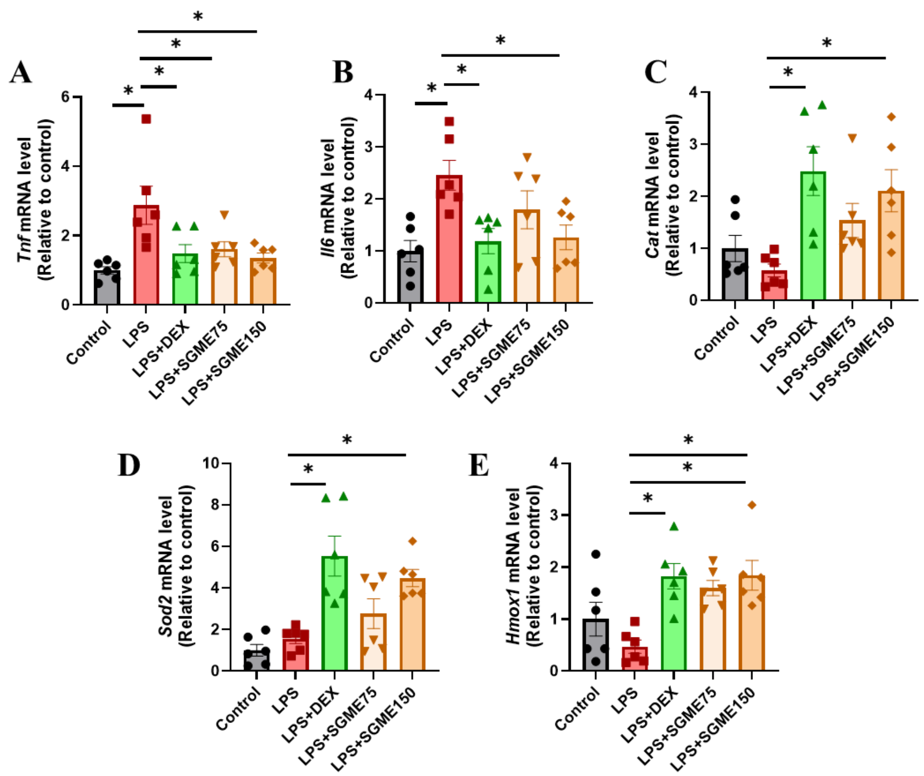

Fig. S4. Effects of SGME on gene expression in lung tissues of LPS-aspirated mice. Mice were treated with SGME at doses of 75 and 150 mg/kg or dexamethasone (1 mg/kg). Then, pulmonary inflammation was induced by administering of LPS (250 µg/mouse) via oropharyngeal inhalation. After 24 h of LPS inhalation, lung tissues were collected for further analyses. (**A-E**) Total RNA was extracted from lung tissues and used to examine mRNA expression of *Tnf* (A), *Il6* (B), *Cat* (C), *Sod2* (D), and *Hmox1* (E) by RT-qPCR analysis. * denotes $p < 0.05$; n = 6.

Figure S5

A

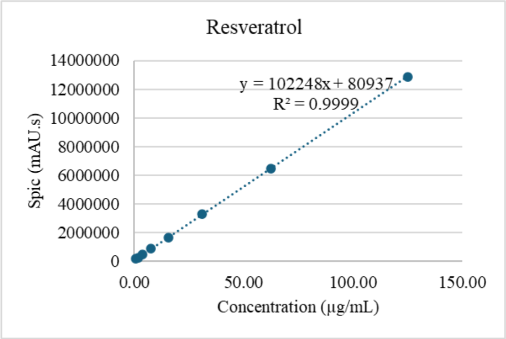

B

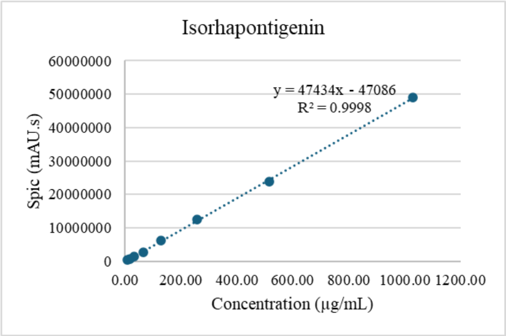

C

Sample	resveratrol content (%)	Isorhapontigenin content (%)
**SGME (n=3)**	0.13 ± 0.00	0.56 ± 0.02
**LOD**	0.002	0.006
**LOQ**	0.008	0.019

Fig. S5. Quantification of stilbenes in SGME. (**A and B**) Calibration curves for resveratrol and isorhapontigenin were generated using the corresponding reference standards. (**C**) The contents of resveratrol and isorhapontigenin in SGME were expressed as % (w/w). Data were presented as mean ± SE from three independent experiments.

Full blot images for western blot experiments

Figure 1G

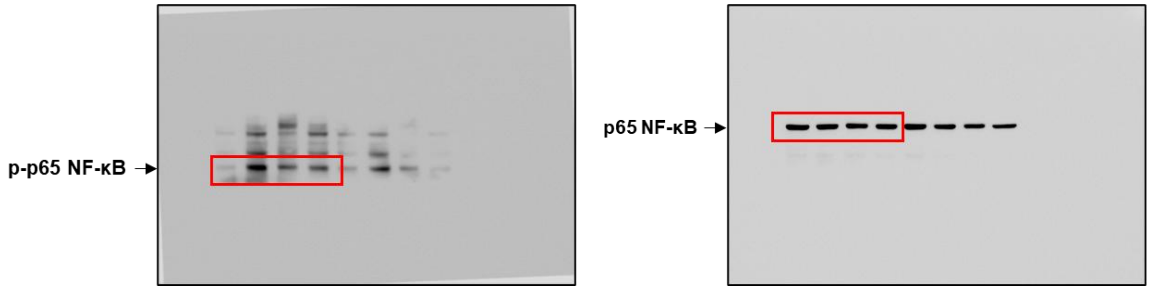

Figure 1H

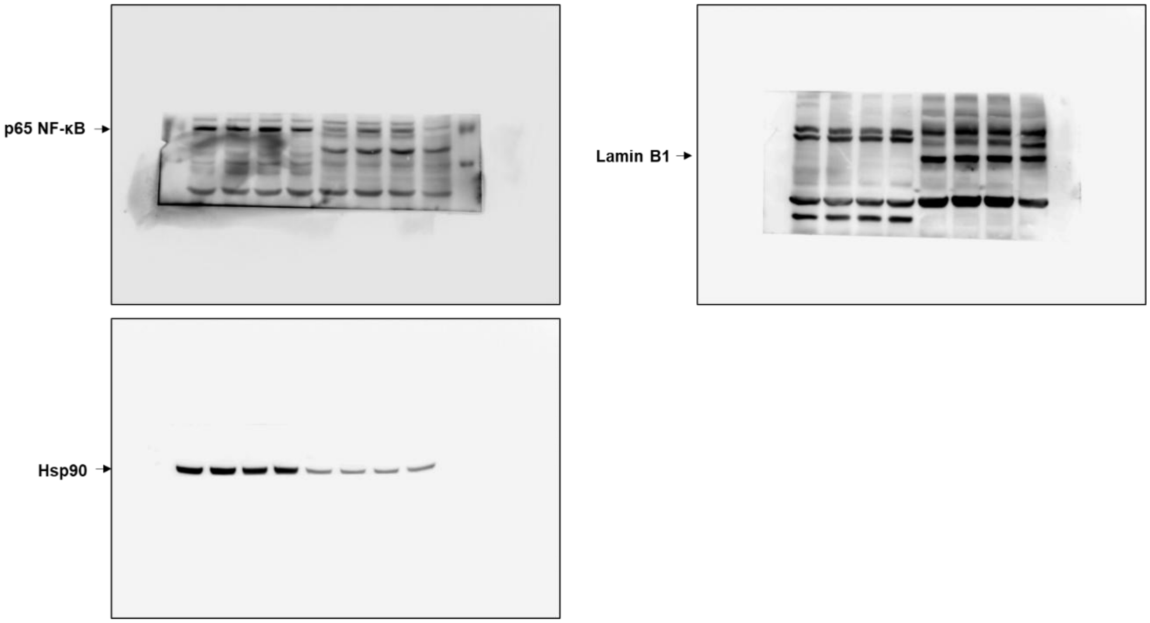

Figure 3A

Nrf2 →

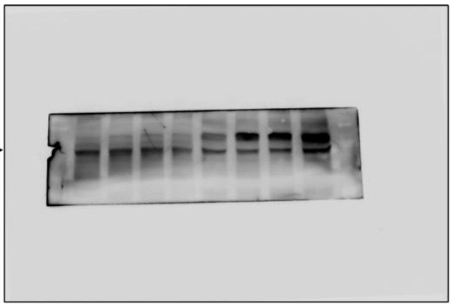

Hsp90 →

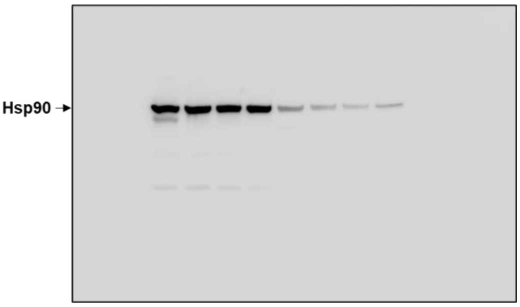

Lamin B1 →

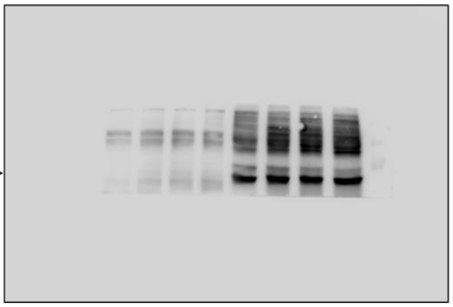

Figure 3E

HO-1 →

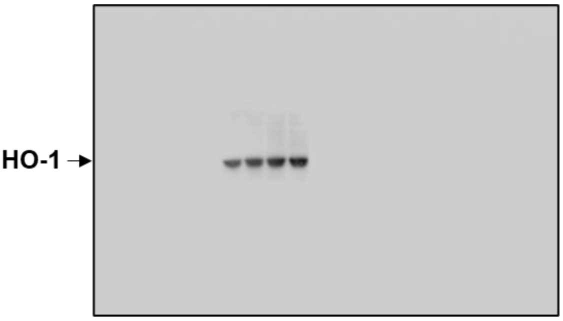

β-actin →

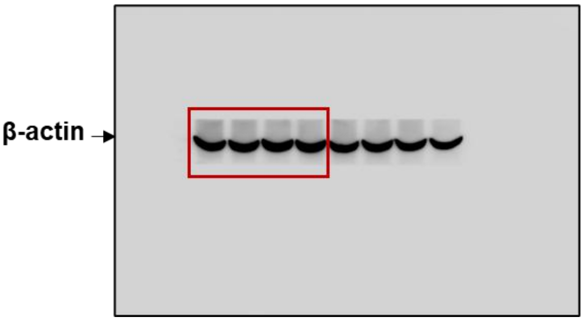

Figure 4D

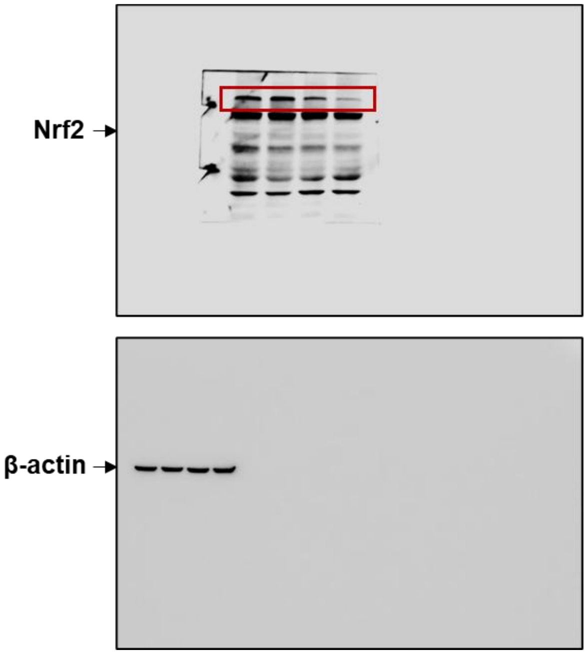

Figure 5G

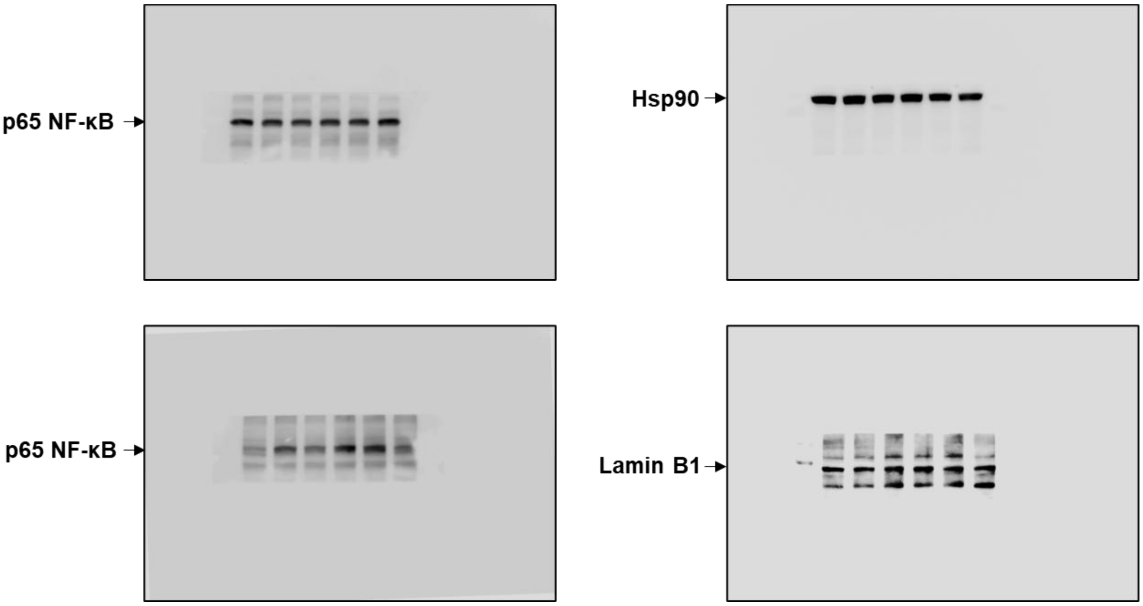

Supplement: Supplementary file 1 — Supplementary Material 1 [file 41598_2026_50370_MOESM1_ESM.pdf]
